# Systemic iron status and maternal pregnancy complications: A Mendelian randomization study

**DOI:** 10.1093/ije/dyac037

**Published:** 2022-06-13

**Authors:** Tormod Rogne, Stephen Burgess, Dipender Gill

**Affiliations:** 1 Department of Chronic Disease Epidemiology and Center for Perinatal, Pediatric and Environmental Epidemiology, Yale School of Public Health, New Haven, CT, USA; 2 Gemini Center for Sepsis Research, Department of Circulation and Medical Imaging, NTNU, Norwegian University of Science and Technology, Trondheim, Norway; 3 Centre for Fertility and Health, Norwegian Institute of Public Health, Oslo, Norway; 4 MRC Biostatistics Unit, University of Cambridge, Cambridge, UK; 5 Cardiovascular Epidemiology Unit, Department of Public Health and Primary Care, University of Cambridge, Cambridge, UK; 6 Clinical Pharmacology and Therapeutics Section, Institute of Medical and Biomedical Education and Institute for Infection and Immunity, St George’s, University of London, London, UK; 7 Department of Epidemiology and Biostatistics, School of Public Health, Imperial College London, London, UK

**Keywords:** Mendelian randomization, iron status, pregnancy complications

## Introduction

Iron deficiency is the most common nutritional deficiency worldwide, and is estimated to affect a quarter of the pregnant population.^1^ Low systemic iron levels have been associated with several maternal pregnancy complications, including infection and preeclampsia.^2^ However, these results from traditional observational studies may be affected by confounding. A systematic review of trials found that daily iron supplementation in pregnancy reduced iron deficiency anemia, but there was no clear effect on other maternal complications, which in large part may be due to low statistical power.^3^

To robustly evaluate whether systemic iron status affects risk of maternal pregnancy complications, we conducted a Mendelian randomization (MR) study. By leveraging genetic instruments for systemic iron status, the approach greatly reduced the risk of confounding and allowed for a large study sample.

## Methods

In this two-sample MR study, we evaluated the association between genetically-predicted iron homeostasis biomarkers and the following five maternal pregnancy complications: abruptio placenta, genitourinary tract infection, postpartum hemorrhage, premature rupture of membranes, and preeclampsia.

The genetic instruments for the iron homeostasis biomarkers were collected from a recent genome-wide association study of 84,328 premenopausal women (Supplementary Methods).^4^ Our primary exposure of interest was systemic iron status; measured through a composite of multiple iron homeostasis biomarkers.^5^ As our instruments for systemic iron status we therefore used the four uncorrelated single-nucleotide polymorphisms that were robustly associated with each of serum iron, iron binding capacity, transferrin and ferritin, as has previously been done.^5^ In sensitivity analyses, we conducted separate analyses for each of the four iron homeostasis biomarkers, using all uncorrelated genetic variants robustly associated with the respective biomarker.

The genetic associations with the outcomes of interest were collected from relevant genome-wide association studies (Supplementary Methods).

The main analysis was the inverse-variance weighted method, which assumes all genetic instruments to be valid.^6^ MR estimates may be biased by pleiotropy (the genetic instruments affect the outcome other than through the exposure) and weak instruments: Weighted mode, weighted median, and MR Egger sensitivity analyses were conducted to address the former,^6^ while weak instruments were accounted for using robust adjusted profile scores.^6^ To account for multiple testing, we used p-value < 0.01 as threshold for statistical significance. All MR analyses were conducted using the TwoSampleMR package (version 0.5.6) in R (version 3.6.2).

Publicly available data with relevant ethical approvals were used.

## Results

Genetically-predicted systemic iron status was significantly associated with risk of genitourinary tract infection in pregnancy; odds ratio 0.65 (95% confidence interval 0.47 to 0.89, p-value = 0.0085) for each standard deviation increase in serum iron (Figure). There was a tendency of a protective effect of increasing genetically-predicted systemic iron status on abruptio placenta, but low precision yielded inconclusive results. Genetically-predicted systemic iron status was not clearly linked to the three other complications.

MR sensitivity analyses supported the findings from the main analyses (Figure), as did the sensitivity analyses of transferrin saturation and serum iron (Table).

Finally, we conducted a *post hoc* analysis of genetically-predicted systemic iron status and risk of urinary tract infection among non-pregnant women (Supplementary Methods) which yielded no strong association (inverse-variance weighted analysis; odds ratio 1.00, 95% confidence interval 0.69 to 1.46).

## Discussion

We found evidence supporting a protective effect of increasing systemic iron status on risk of genitourinary tract infection in pregnancy. Considering this finding together with previous observations,^2^ it is plausible that the reported association is causal. Given the increased demands of iron in pregnancy, and that a quarter of the pregnant population are iron deficient,^1^ our findings support efforts in public health and antenatal care to ensure adequate iron status among pregnant women.

## Figures and Tables

**Figure:**
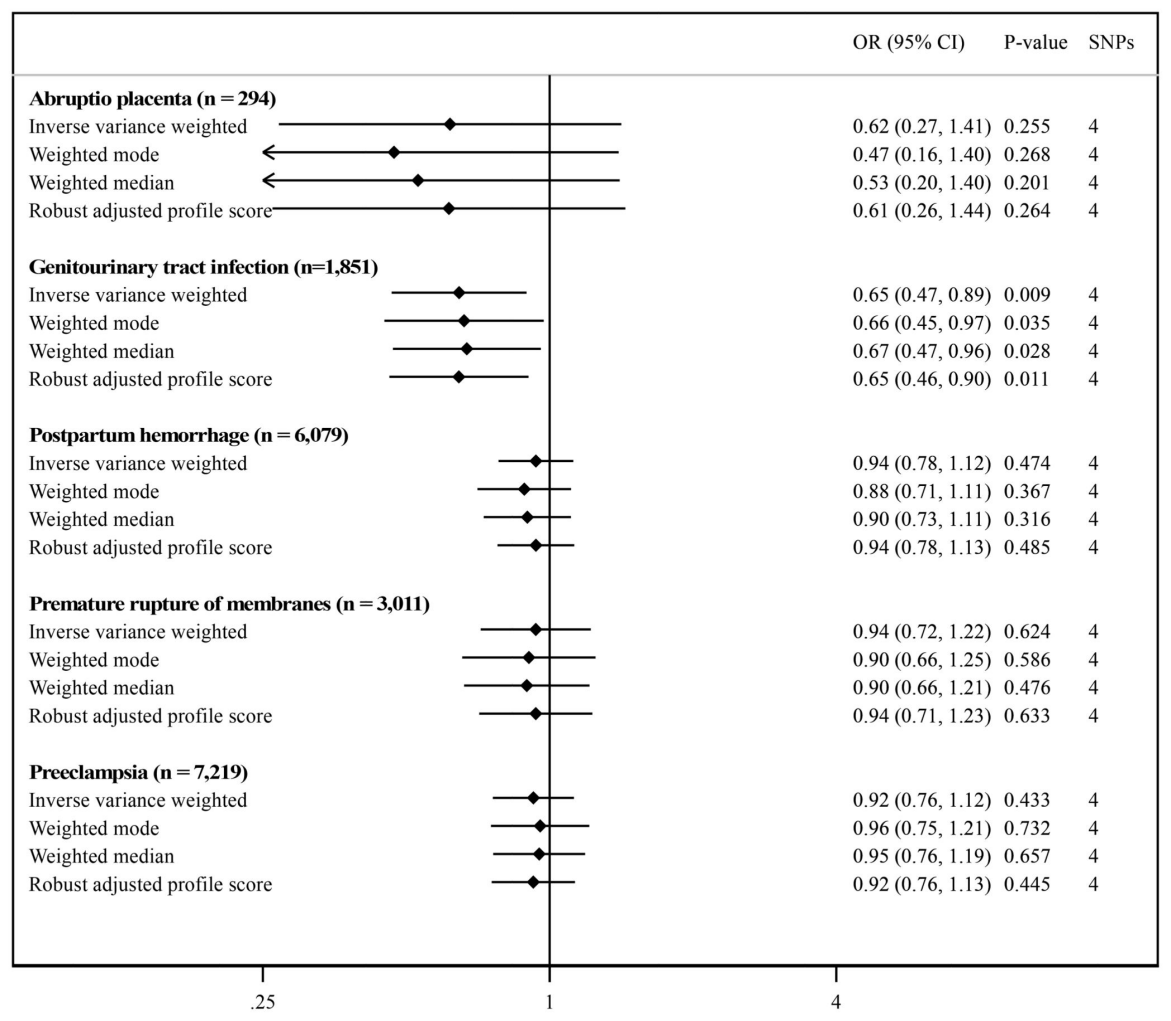
Systemic iron status and risk of maternal pregnancy complications. Unit of exposure is one standard deviation (7.1 μmol/L) increase of genetically-predicted serum iron using the four uncorrelated genetic instruments from the three genes (*DUOX2*, *HFE*, and *TMPRSS6*) linked to all iron homeostasis biomarkers.^4^ The number of cases are reported for each complication (number of controls presented in Supplementary Methods). CI, confidence interval; OR, odds ratio; SNPs, single-nucleotide polymorphisms.

**Table:** Sensitivity analysis of iron homeostasis biomarkers and risk of maternal pregnancy complications.

	Abruptio placenta		Genitourinary tract infection		Postpartum hemorrhage		Premature rupture of membranes		Preeclampsia	
	OR (95% CI)	P-value	OR (95% CI)	P-value	OR (95% CI)	P-value	OR (95% CI)	P-value	OR (95% CI)	P-value
**Transferrin saturation**										
Inverse variance weighted	0.53 (0.21-1.28)	0.161	0.60 (0.42-0.84)	0.003	0.94 (0.78-1.13)	0.558	0.93 (0.70-1.24)	0.644	0.84 (0.69-1.02)	0.093
Weighted mode	0.36 (0.12-1.06)	0.101	0.63 (0.42-0.95)	0.029	0.99 (0.78-1.25)	0.942	0.90 (0.65-1.23)	0.541	0.88 (0.71-1.09)	0.297
Weighted median	0.37 (0.12-1.10)	0.076	0.67 (0.43-1.02)	0.067	0.97 (0.77-1.21)	0.810	0.85 (0.59-1.22)	0.396	0.89 (0.70-1.13)	0.353
MR Egger	0.58 (0.16-2.05)	0.427	0.65 (0.40-1.06)	0.126	0.98 (0.75-1.28)	0.915	0.93 (0.62-1.40)	0.752	0.85 (0.64-1.12)	0.293
Robust adjusted profile score	0.53 (0.21-1.34)	0.183	0.59 (0.41-0.85)	0.005	0.94 (0.77-1.14)	0.560	0.93 (0.69-1.25)	0.632	0.85 (0.69-1.04)	0.127
**Iron**										
Inverse variance weighted	0.55 (0.25-1.17)	0.124	0.62 (0.45-0.84)	0.002	0.94 (0.79-1.10)	0.474	0.95 (0.74-1.21)	0.712	0.90 (0.76-1.07)	0.265
Weighted mode	0.46 (0.18-1.17)	0.132	0.66 (0.46-0.93)	0.015	0.92 (0.76-1.12)	0.443	0.94 (0.71-1.23)	0.670	0.94 (0.77-1.15)	0.604
Weighted median	0.50 (0.19-1.32)	0.164	0.66 (0.45-0.97)	0.035	0.89 (0.72-1.09)	0.275	0.89 (0.66-1.20)	0.471	0.94 (0.76-1.17)	0.611
MR Egger	0.67 (0.21-2.07)	0.506	0.66 (0.41-1.06)	0.115	0.91 (0.71-1.16)	0.487	1.02 (0.71-1.47)	0.889	0.85 (0.65-1.11)	0.281
Robust adjusted profile score	0.54 (0.25-1.19)	0.131	0.61 (0.45-0.83)	0.002	0.93 (0.79-1.11)	0.452	0.95 (0.74-1.23)	0.727	0.91 (0.76-1.09)	0.346
**Iron binding capacity**										
Inverse variance weighted	0.98 (0.67-1.43)	0.922	1.06 (0.92-1.23)	0.388	1.00 (0.92-1.09)	0.915	1.05 (0.93-1.18)	0.409	1.05 (0.95-1.17)	0.293
Weighted mode	0.98 (0.64-1.48)	0.927	1.05 (0.89-1.23)	0.561	0.98 (0.90-1.07)	0.753	1.06 (0.93-1.21)	0.365	1.03 (0.94-1.14)	0.459
Weighted median	1.00 (0.65-1.55)	0.980	1.00 (0.84-1.18)	0.994	0.97 (0.89-1.07)	0.630	1.04 (0.91-1.20)	0.506	1.04 (0.94-1.15)	0.428
MR Egger	0.94 (0.58-1.53)	0.825	1.04 (0.86-1.26)	0.639	0.98 (0.88-1.10)	0.824	1.02 (0.87-1.19)	0.780	1.02 (0.89-1.17)	0.749
Robust adjusted profile score	0.98 (0.66-1.44)	0.924	1.06 (0.91-1.23)	0.443	0.99 (0.91-1.08)	0.988	1.05 (0.92-1.19)	0.427	1.05 (0.94-1.16)	0.361
**Ferritin**										
Inverse variance weighted	1.74 (0.75-4.01)	0.191	0.87 (0.61-1.24)	0.457	0.92 (0.77-1.10)	0.376	1.08 (0.84-1.39)	0.517	0.82 (0.64-1.05)	0.119
Weighted mode	1.35 (0.43-4.23)	0.608	0.68 (0.41-1.12)	0.148	0.87 (0.65-1.17)	0.385	0.99 (0.67-1.45)	0.962	0.85 (0.65-1.11)	0.250
Weighted median	1.13 (0.37-3.41)	0.820	0.69 (0.43-1.13)	0.149	0.96 (0.72-1.28)	0.813	1.09 (0.76-1.56)	0.622	0.79 (0.59-1.06)	0.121
MR Egger	0.48 (0.14-1.64)	0.257	0.76 (0.43-1.34)	0.358	0.84 (0.63-1.11)	0.247	0.99 (0.67-1.47)	0.990	0.81 (0.55-1.20)	0.317
Robust adjusted profile score	1.70 (0.72-4.04)	0.224	0.86 (0.61-1.21)	0.408	0.93 (0.77-1.12)	0.472	1.08 (0.83-1.40)	0.550	0.83 (0.66-1.04)	0.107

Estimates represent odds ratios per standard deviation increase in each exposure, which is 11.7 %, 7.1 μmol/L, 14.9 μmol/L, and 70.5 μg/L for transferrin saturation, serum iron, iron binding capacity and ferritin, respectively. The following maximum number of single-nucleotide polymorphisms were included in the analyses: Transferring saturation, n = 10; iron, n = 14; iron binding capacity, n = 16; and ferritin, n = 37. CI, confidence interval; OR, odds ratio
